# Employing deep learning and transfer learning for accurate brain tumor detection

**DOI:** 10.1038/s41598-024-57970-7

**Published:** 2024-03-27

**Authors:** Sandeep Kumar Mathivanan, Sridevi Sonaimuthu, Sankar Murugesan, Hariharan Rajadurai, Basu Dev Shivahare, Mohd Asif Shah

**Affiliations:** 1 School of Computer Science and Engineering, Galgotias University, Greater Noida, 203201 India; 2 Department of Computer Science and Engineering, Vel Tech Rangarajan Dr.Sagunthala R&D Institute of Science and Technology, Chennai, 600062 India; 3 School of Computing Science and Engineering, VIT Bhopal University, Bhopal–Indore Highway Kothrikalan, Sehore, 466114 India; 4 Kebri Dehar University, 250, Kebri Dehar, Somali Ethiopia; 5 Centre of Research Impact and Outcome, Chitkara University Institute of Engineering and Technology, Chitkara University, Rajpura, Punjab 140401 India; 6 Division of Research and Development, Lovely Professional University, Phagwara, Punjab 144001 India

**Keywords:** Artificial intelligence, Brain tumor, Transfer learning, Diagnosis, Medical imaging, Cancer, Health care, Medical research, Neurology

## Abstract

Artificial intelligence-powered deep learning methods are being used to diagnose brain tumors with high accuracy, owing to their ability to process large amounts of data. Magnetic resonance imaging stands as the gold standard for brain tumor diagnosis using machine vision, surpassing computed tomography, ultrasound, and X-ray imaging in its effectiveness. Despite this, brain tumor diagnosis remains a challenging endeavour due to the intricate structure of the brain. This study delves into the potential of deep transfer learning architectures to elevate the accuracy of brain tumor diagnosis. Transfer learning is a machine learning technique that allows us to repurpose pre-trained models on new tasks. This can be particularly useful for medical imaging tasks, where labelled data is often scarce. Four distinct transfer learning architectures were assessed in this study: ResNet152, VGG19, DenseNet169, and MobileNetv3. The models were trained and validated on a dataset from benchmark database: Kaggle. Five-fold cross validation was adopted for training and testing. To enhance the balance of the dataset and improve the performance of the models, image enhancement techniques were applied to the data for the four categories: pituitary, normal, meningioma, and glioma. MobileNetv3 achieved the highest accuracy of 99.75%, significantly outperforming other existing methods. This demonstrates the potential of deep transfer learning architectures to revolutionize the field of brain tumor diagnosis.

## Introduction

The human brain, located in the cranium, is a crucial organ responsible for various functions, governed by a network of billions of neurons that coordinate electrical and chemical impulses, shaping our experiences and existence^1^. This extraordinary organ is a linchpin in the realms of perception, emotion, and character. Comprising distinct components, each with specialized roles, the brain epitomizes complexity. The cerebral cortex, a convoluted outer layer, takes the reins of consciousness, while the cerebellum assumes responsibility for balance and coordination^2^. This harmonious collaboration among various brain regions is crucial for the seamless orchestration of our daily activities and responses to the world around us. However, the resilient nature of the brain does not shield it entirely from threats. The emergence of abnormal cell growth, encapsulated as a mass or lump, is known as a tumor or neoplasm^3^. Tumors can be found in various organs, including the brain. The distinction between benign and malignant tumors is crucial for understanding their health impact. Benign tumors, slow and localized, are less dangerous but can pose a threat if they encroach on vital organs or tissues^4^. Malignant tumors are aggressive and can invade surrounding tissues and spread through metastasis. Understanding the growth and behavior of these tumors is crucial for timely intervention and preserving the intricate functionality of the human brain, which is a marvel that weaves the tapestry of human experience^5^. A brain tumor is an abnormal cell accumulation within the brain, which can either emerge directly from brain tissue or infiltrate the brain through metastasis, where cancerous cells from other parts of the body spread to the brain^6^. Brain tumor diagnosis involves a comprehensive approach, often involving imaging tests and a biopsy to identify the tumors characteristics and grade. The diverse spectrum of brain tumors includes neoplasms from various cell types, each with unique challenges and implications that influence diagnostic approaches, treatment strategies, and patient outcomes^7^. Malignant gliomas, arising from brain's glial cells, can develop in any brain region and require targeted therapeutic interventions to navigate the cellular matrix, emphasizing the need for effective treatment in this formidable tumor type^8^. Meningiomas, a distinct category of tumors, originate in the meninges, the protective membranes enveloping the brain and spinal cord. Interestingly, most meningiomas are relatively benign and often do not pose an immediate threat to health^9^. The pituitary gland, located at the brain's base, can cause adenomas, tumors disrupting hormonal regulation, and Schwannomas, stemming from Schwann cells responsible for creating the myelin sheath that protects nerve fibers^10^. Glioblastomas, the most malignant and aggressive type of brain tumors, pose significant challenges in diagnosis and treatment. Understanding the intricacies of these diverse brain tumors is crucial for tailoring effective treatment strategies, adding complexity to the understanding of brain pathology^11^.

The relentless pursuit of knowledge in neuro-oncology holds the promise of advancing diagnostic techniques and therapeutic interventions, providing a beacon of hope for individuals grappling with the complexities of these formidable intrusions into the delicate domain of the human brain^12^. The integration of deep learning and artificial intelligence (AI) has significantly improved medical image analysis, leading to significant advancements in the detection, diagnosis, and characterization of various medical conditions. This has enabled healthcare professionals to make more informed decisions, particularly in the accurate classification of cancer types, such as lung and breast cancer. This integration has resulted in earlier diagnoses, improved treatment decisions, and improved patient outcomes^13^. Artificial intelligence plays a crucial role in surgical planning, enabling precise segmentation of lesion boundaries and brain structures, balancing intervention with quality-of-life preservation. It predicts complications, recurrence rates, and therapeutic responses, guiding optimal follow-up strategies and enabling personalized patient guidance through tailored screening protocols^14^. Transfer learning (TL) is a machine learning technique that has gained significant attention in the medical field, focusing on leveraging pre-existing models trained on large datasets for specific tasks^15^. Transfer learning is a crucial tool in medical image analysis, enabling the creation of high-performing models with reduced training time and computational cost. As the field evolves, transfer learning is expected to play a more significant role in improving patient care. Various transfer learning models, including VGG, ResNet, Inception, MobileNet, and DenseNet, have shown remarkable efficacy in this area^16^. Transfer learning models, utilizing neural networks' depth and complexity, are used to identify intricate patterns in medical images. This versatile approach extends beyond these well-known architectures, with numerous other models contributing to the growing range of tools for medical imaging analysis^17^. Transfer learning in medical imaging has significantly expedited the development process and improved the performance and accuracy of pre-trained models, enabling faster and more accurate diagnoses of cancerous lesions, particularly in the identification and classification of cancerous lesions^18^. The efficiency gains achieved through transfer learning models have significant implications for patient care, as early detection and precise classification of cancer types are essential for initiating timely and targeted treatment strategies. As the synergy between deep learning, artificial intelligence, and transfer learning continues to evolve, the landscape of medical image analysis is poised for transformative change. The amalgamation of these technologies not only augments the capabilities of healthcare professionals but also holds the promise of improving patient outcomes and reshaping the paradigm of medical diagnostics. In our study, we compared four transfer learning models—VGG19, ResNet152, DenseNet169, and MobileNetv3—to determine which one is most effective in classifying brain MRI data. The main contribution of our paper lies in the innovative use of transfer learning and fine-tuning on MR images to categorize brain tumors into four groups.

(i) We fine-tuned the transfer learning models after processing and applied them to three benchmark datasets to optimize their performance. Additionally, we enhanced models like ResNet152, VGG19, DenseNet169, and MobileNetv3 by adding a single fully connected layer.
(ii) To establish a meaningful comparison, we created a benchmark against which our proposed transfer learning methodologies can be evaluated in comparison to previous research. The key outcome of our study is the achievement of maximum precision. MobileNetv3 demonstrated outstanding precision of 99.75% in a historical context, while InceptionV3 achieved remarkable precision of 98.8% in operational scenarios.
(iii) Transfer learning allows leveraging pre-trained models, especially beneficial when dealing with limited labelled medical data.
(iv) MobileNetv3, a specific transfer learning architecture, achieved exceptional accuracy in brain tumor diagnosis.
(v) These results highlight the effectiveness of our transfer learning methodologies in the classification of brain tumors, showcasing their potential impact on advancing diagnostic accuracy in medical image analysis.

The article is structured as follows: Section "Related work": Provides a concise overview of the relevant literature. Section "Material and methods": Introduces the proposed methodology and outlines the experimental setup, including data preparation, model training, and performance evaluation. Section "Experimental results and discussion": Presents the experimental results and their thorough analysis. Section "Conclusion and future work": Concludes the article with a summary of the findings and outlines potential directions for future research.

## Related work

Leveraging the power of deep convolutional neural networks, we developed a highly accurate framework for classifying brain tumors into three distinct categories: meningioma, glioma, and pituitary adenoma. Our proposed approach employs three different CNN architectures, namely AlexNet, GoogLeNet, and VGGNet, to extract relevant and robust features from MRI scans. To further enhance the performance of our models, we employed transfer learning strategies, including fine-tuning and freezing, and data augmentation techniques to expand the dataset and reduce overfitting. Extensive experimentation using the Figshare MRI brain tumor dataset revealed that the optimized VGG16 architecture achieved an impressive detection and classification accuracy of up to 98.69%, demonstrating the effectiveness of our proposed framework in accurate brain tumor categorization^19^. In this study, a probabilistic neural network (PNN) is employed for classifying MR brain images. PNN is chosen due to its simple structure and rapid training process. A dataset of 30 brain MRI samples was used to train the PNN classifier, and its performance was evaluated using 12 different sets of images. The trained classifier was tested with a range of smoothing factors, including spread. Experimental results demonstrate that the PNN classifier achieves an accuracy of 83.3%, which is considered effective given the spread value^20^. The proposed method employs a three-step structure for improved clarity. Initially, contextual information is incorporated by enhancing the tumour region and designating it as the region of interest. Subsequently, an adaptive spatial division algorithm, grounded in intensity ordering, partitions the expanded tumour region into subregions. Raw image patches, serving as local characteristics, are then extracted from these subregions. In the final step, the Fisher kernel framework is employed to amalgamate the local characteristics of each subregion into a singular vector representation. Concatenating these representations results in the creation of an image-level signature. Subsequently, the comparison between the query picture and the images stored in the database is carried out using a closed-form metric learning method after extracting features. The evaluation, performed on a substantial dataset consisting of 3604 images featuring meningiomas, gliomas, and pituitary tumours, demonstrates an impressive 94.68% average accuracy in extensive studies^21^.

Early signs of Parkinson's disease (PD) can be detected in a person's handwriting. Leveraging transfer learning and data augmentation strategies, this study introduces a novel deep convolutional neural network (CNN) classifier for accurate PD diagnosis. Two transfer learning methods, freezing and fine-tuning, are evaluated using the ImageNet and MNIST datasets as source tasks. A fine-tuning-based strategy applied to the ImageNet and PaHaW datasets resulted in a trained network with an accuracy of 98.28%^22^. This study utilizes an advanced deep learning technique to identify and classify brain tumors in MRI scans. Diagnosing brain tumors, a critical task, is time-consuming and labor-intensive for radiologists. Their assessments are solely based on their expertise and individual judgments, which are often inaccurate. To address the growing challenge of accurate brain tumor diagnosis, this work employs deep learning to categorize brain tumor MRI images with high precision. AlexNet's convolutional neural network (CNN) transfer learning model was employed for this purpose. Our technology streamlines the entire diagnostic process, achieving an accuracy of 99.62%, thereby enhancing resilience, efficiency, and accuracy in healthcare^23^. The integration of artificial intelligence (AI), specifically leveraging deep learning (DL), into medical imaging has transformed the landscape of classifying and detecting intricate medical conditions, such as brain tumors and other serious diseases. Deep learning has showcased exceptional proficiency in accurately segmenting and classifying brain tumors. This study introduces an AI-driven methodology for the classification of brain tumors, employing deep learning algorithms and utilizing publicly available datasets. These datasets categorize brain tumors into two groups: malignant and noncancerous, comprising a testing set of 696 T1-weighted images. The proposed approach attains notable performance, achieving a maximum accuracy of 99.04%. These outcomes underscore the efficacy of the proposed algorithm in the precise classification of brain tumor^24^. This study aims to automate the detection and diagnosis of brain tumors through the implementation of a fine-grained classification technique. The performance of nine pre-trained transfer learning (TL) classifiers—namely, InceptionResNetV2, InceptionV3, Xception, ResNet18, ResNet50, ResNet101, ShuffleNet, DenseNet201, and MobileNetV2—is systematically compared. The evaluation utilizes a publicly available brain tumor classification (MRI) dataset sourced from Kaggle. Notably, the InceptionResNetV2 TL method outperforms other deep learning (DL) techniques, achieving impressive accuracy (98.91%), precision (98.28%), recall (99.75%), and F-measure (99%) values^25^. Embracing a multilayer-based metadata learning strategy and incorporating a convolutional neural network (CNN) layer, the proposed system architecture facilitates accurate brain MRI classification. To effectively handle high-dimensional data, sparse coding estimates are employed, while metadata-based vector encoding serves as the encoding scheme. This innovative approach yields results that are both objectively and subjectively compelling in terms of categorization. Validated using two datasets, BRATS and REMBRANDT, the proposed brain MRI classification algorithm surpasses the performance of existing methods^26^. Employing a multi-stage approach, the proposed method commences with preprocessing MRI images to eliminate noise and artifacts using an adaptive filter. Subsequently, enhanced fuzzy c-means clustering (EFCMC) is applied for image segmentation, followed by feature extraction utilizing the local-binary grey level co-occurrence matrix (LBGLCM). This comprehensive strategy achieves remarkable classification performance, attaining a sensitivity of 98.79%, a specificity of 91.3%, and an accuracy of 98.1% in brain tumor classification^27^.

Kirsch's edge detectors are utilized to identify boundary edge pixels, followed by contrast adaptive histogram equalization to enhance the brain image. Subsequently, the enhanced brain image is transformed using Ridgelet transform to obtain multi-resolution coefficients. Features are extracted from Ridgelet transformed coefficients, optimized using PCA, and classified as Glioma or non-Glioma using the Co-Active Adaptive Neuro Fuzzy Expert System (CANFES) classifier. This comprehensive methodology achieves remarkable classification performance, attaining 97.6% sensitivity, 98.56% specificity, 98.73% accuracy, 98.85% precision, 98.11% FPR, and 98.185 FNR. While images can enhance the content, they are not always necessary. In this case, the revised sentence provides a clear and concise description of the proposed methodology and its performance without the need for visual aids^28^. This study presents a novel brain tumor classification method using deep transfer learning, incorporating a new fine-tuning technique and an SVM classifier. The proposed transfer learning-based classification strategy is evaluated on the Figshare dataset, which includes MRI brain tumors of meningioma, glioma, and pituitary gland origin, under various scenarios. The proposed deep transfer learning approach demonstrates promising results, achieving 99.35% accuracy with a CNN architecture and an SVM classifier, and 99.61% accuracy with a ResNet-50 architecture and fine-tuning parameters^29^. A lightweight ensemble model has been developed to improve brain cancer detection and classification using MRI data. The model incorporates MRI preprocessing, intensity, texture, and shape feature extraction. The model was evaluated using the BraTS 2020 dataset and achieved excellent performance, with 93.0% accuracy, 0.94 precision, 0.93 recall, 0.94 F1 score, and an AUC-ROC value of 0.984. This approach offers a valuable tool for early diagnosis and effective treatment planning in brain cancer^30^. The solution for brain tumor segmentation in medical imaging, it uses the U-Net model architecture, known for its semantic segmentation performance, to train models on distributed data from various medical institutions. The federated learning approach is scalable, suitable for large-scale deployment in medical imaging. The experimental results show a significant improvement in specificity and dice coefficient when increasing the number of clients. The method surpasses existing CNN and RNN-based approaches, achieving higher accuracy, performance, and efficiency. The findings hold promise for wider adoption in medical imaging applications without compromising data confidentiality^31^. A hybrid methodology for brain tumor segmentation in MRI scans, combining handcrafted features with convolutional neural networks. The approach extracts feature from MRI scans and trains a CNN architecture to detect relevant data. The Brain Tumor Segmentation challenge dataset evaluated the performance of the hybrid approach, showing superior performance compared to conventional methods. The research holds promise for real-world clinical applications^32^. A cascaded strategy for brain tumor segmentation, integrating convolutional neural networks (CNNs) with handcrafted feature-based machine learning algorithms. The method uses data from four MRI modalities and a Global Convolutional Neural Network (GCNN). The model achieved a Dice score of 87%, surpassing state-of-the-art methods. This innovative approach has the potential to significantly enhance brain tumor segmentation, aiding clinicians in diagnosing and treating patients, and reducing the cost, time, and error of manual segmentation^33^. Table 1 provides a comprehensive overview of the different state-of-the-art methods that have been incorporated into our proposed model.

**Table 1:** State-of-the-art methods details.

Author	Year	Dataset	Method	Limitations
Arshia Rehman^18^	2019	Figshare	AlexNet, GoogLeNet, VGGNet	Absence of an in-depth analysis or explanation of the interpretability of the model
Tasnim Azad Abir^19^	2018	Kaggle	PNN	Lack of detailed analysis or discussion regarding the potential biases present in the training data
Jun Cheng^20^	2016	Figshare	Content-based image retrieval	Lack of explicit discussion or consideration of potential limitations related to the generalization of the proposed algorithm to external datasets or diverse clinical settings
Amina Naseer^21^	2019	MNIST, PaHaW	ImageNet	The absence of a detailed discussion or analysis regarding the potential biases present in the training datasets, particularly ImageNet and MNIST, which were used as source tasks for transfer learning
Bakary Badjie^22^	2022	Kaggle	AlexNet's CNN	Lack of explicit consideration or discussion about the interpretability of the deep learning mode
Rajat Mehrotra^23^	2020	Figshare	CNN	The absence of a comprehensive analysis or discussion about the potential impact of class imbalances in the dataset on the model's performance
Naeem Ullah^24^	2022	Kaggle	Inceptionresnetv2	The comparatively weak performance of pre-trained deep learning (DL) models when used as stand-alone classifiers
Saravanan^25^	2022	BRATS, REMBRANDT	CDBLNL	Lack of clarity or detailed discussion regarding the potential limitations or challenges associated with the proposed CDBLNL model
Saravanan Srinivasan^26^	2023	REMBRANDT	Convolutional RNN	One demerit in the presented work is the lack of detailed analysis or discussion about the interpretability of the proposed CRNN (Convolutional Recurrent Neural Network) model
Pshtiwan Jabar Karim^28^	2023	Figshare	CNN + fine-tuned SVM	Lack of detailed discussion or exploration of potential biases in the Figshare dataset used for evaluation. Biases in medical datasets, especially related to brain tumors, can significantly impact the generalizability of the proposed classification method

## Material and methods

### Material

**Figure 1:**
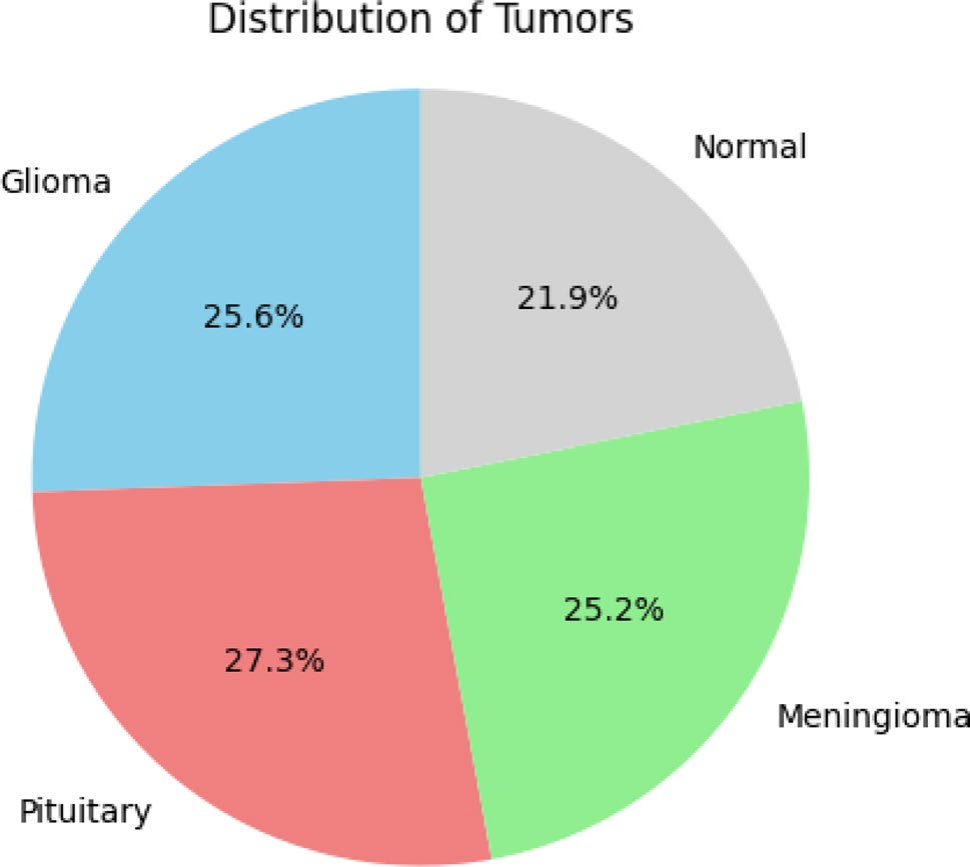
Pie chart illustrating the distribution of images across tumor classes.

For model training, we utilized the brain tumor dataset sourced from Kaggle^34^. This dataset encompasses MRI images of the brains of 7,023 individuals, including those with brain tumors and those without. It comprises cases of meningioma, glioma, pituitary gland tumors, and non-tumor. Each category within this collection contains over 1,600 high-quality images. Table 2 provides a breakdown of the image distribution across the training and test sets. The dataset consists of a total of 7,023 images. Out of these, 5,618 (80%) images are used for training, while 1,405 (20%) images are used for testing. Among these images, 1405 normal, and 5618 are malignant. Figure 1 depicts the frequency of each type of brain tumor imaging. It reveals that there are approximately 1,800 images in the No Tumor class, 1,757 images in the Pituitary class, 1,645 images in the Glioma class, and 1,621 images in the Meningioma class.

**Table 2:** Training and testing dataset for each class.

Phase	Malignant (80%)	Normal (20%)	Total
Train	4494	1124	5618
Test	1124	281	1405
Total	5618	1405	7023

### Methods

**Figure 2:**
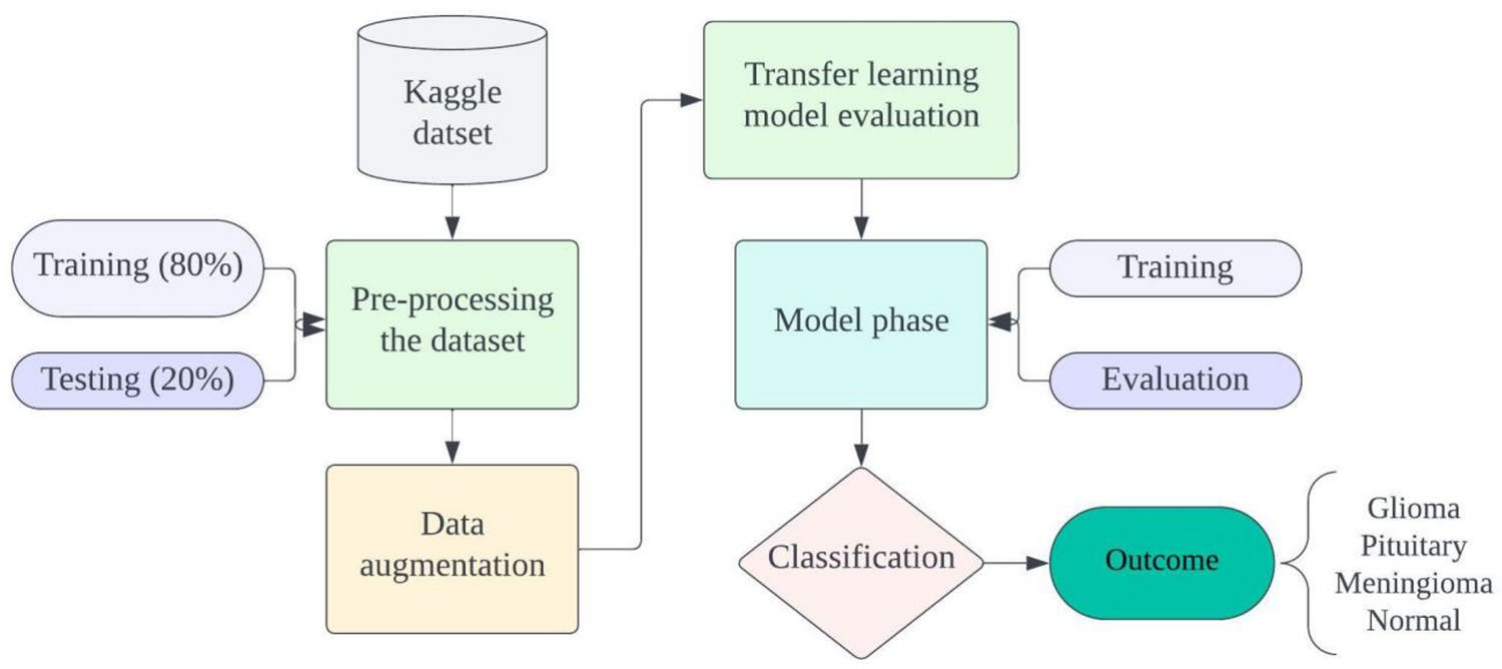
Proposed model architecture.

The proposed model, illustrated in Fig. 2, employs four well-known transfer learning approaches—ResNet152, VGG19, DenseNet169, and MobileNetv3—to create four classes for analyzing and estimating the recommended frame. The data undergoes four transfer learning techniques, and following analysis, it's divided into an 80% training set and a 20% testing set. This split is crucial for training, validating model performance, and assessing generalizability. The proposed model proves reliable in diverse scenarios. In this study, we use image augmentation, a key technique using Keras' ImageDataGenerator, to expand the dataset for training a deep learning model in brain tumor diagnosis. By creating modified copies of images with rotations, zooming, and flipping, the model gets exposed to a wider range of variations, improving its ability to handle new data. This is vital for simulating the variability in medical imaging, making the model more robust to noise and variations. The ultimate goal is to build a reliable and strong deep learning model, especially in the medical field where data is limited, and adapting to diverse and unseen cases is crucial. It introduces variations like rotations, flips, shifts, and zooms, contributing to balanced classes during model training.

**Figure 3:**
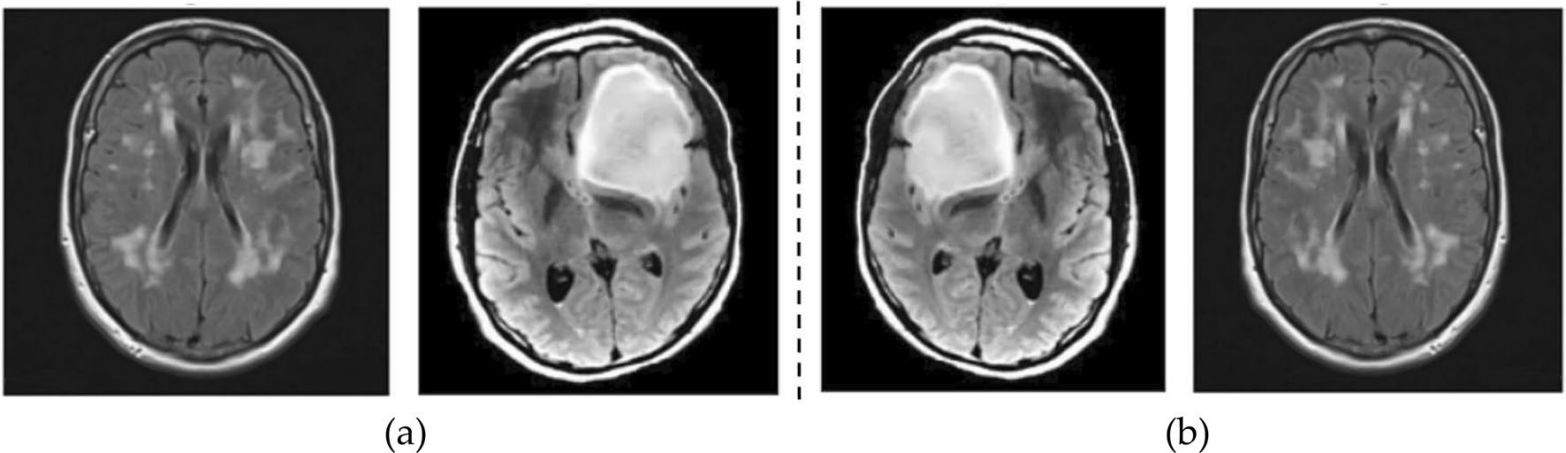
Augmentation (**a**) normal; (**b**) augmented images.

This augmentation strategy contributes to a more comprehensive and diverse training dataset, empowering the model to generalize better across a myriad of scenarios. The utilization of ImageDataGenerator during model training yields a twofold advantage. Firstly, it ensures that the deep learning model is exposed to a richer set of training examples, facilitating improved learning of intricate patterns and features. Secondly, the automatic generation of augmented images enhances the model's robustness by making it less susceptible to overfitting and more adaptable to diverse input variations. This augmentation-driven approach has been recognized for its efficacy in enhancing the overall performance of deep learning models, leading to improved accuracy and resilience in real-world applications^35^. Figure 3 depicts the normal and augmented images of brain MRI.

#### Transfer learning model evaluation

Transfer learning is a machine learning technique that enables a model trained for one task to be repurposed for a different but related task. This method reduces the time and effort required to develop high-performance models, especially for complex tasks like image recognition and natural language processing. By fine-tuning the weights of an existing model, researchers can effectively tackle new challenges. Transfer learning leverages knowledge acquired from a large dataset during initial training, allowing the model to effectively tackle new challenges. This approach contrasts with the traditional method of training a model from the ground up, which can be time-consuming and resource-intensive. Transfer learning has proven successful in various domains, including image identification, natural language processing, and speech recognition, especially in scenarios with limited training data. In this work, four different transfer learning models were employed, each using an input RGB picture size of (224 × 224) to ensure uniformity across all models. Transfer learning has played a crucial role in numerous deep learning applications, including image categorization, object recognition, and medical condition diagnosis.

##### ResNet152

**Figure 4:**
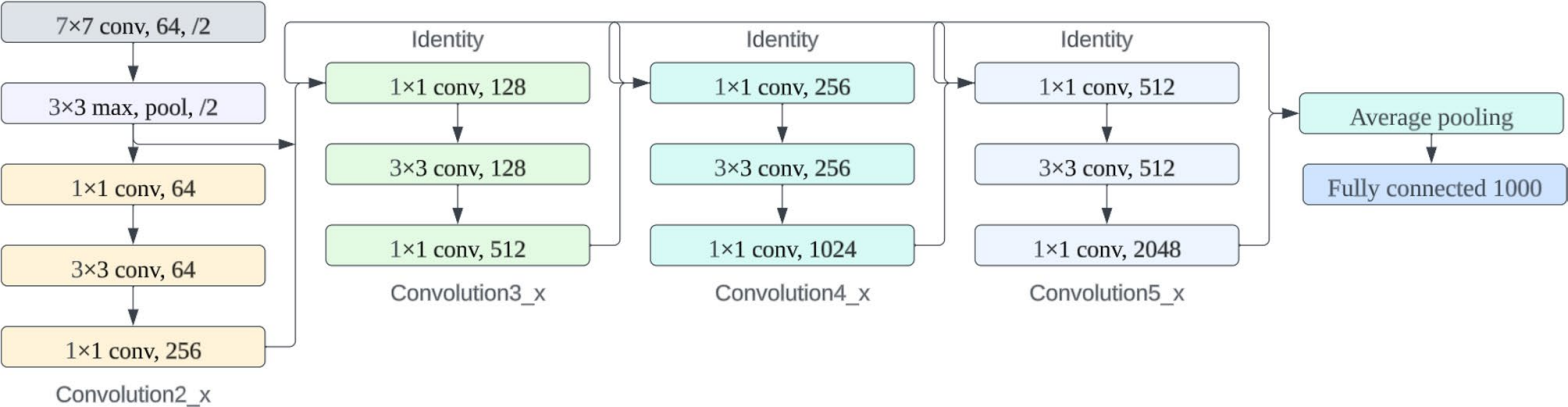
ResNet152 architecture.

ResNet-152 is a deep convolutional neural network architecture developed by Microsoft Research, featuring 152 layers. Its key innovation is the introduction of residual connections or skip connections, which enable the network to learn residual functions, making it easier to train very deep networks^36^. ResNet-152's depth allows it to extract intricate features and patterns from data, making it effective for tasks like image classification and object recognition. This architectural depth, coupled with skip connections, addresses the vanishing gradient problem, facilitating the training of extremely deep networks refer in Fig. 4.

##### Visual geometry group 19

**Figure 5:**
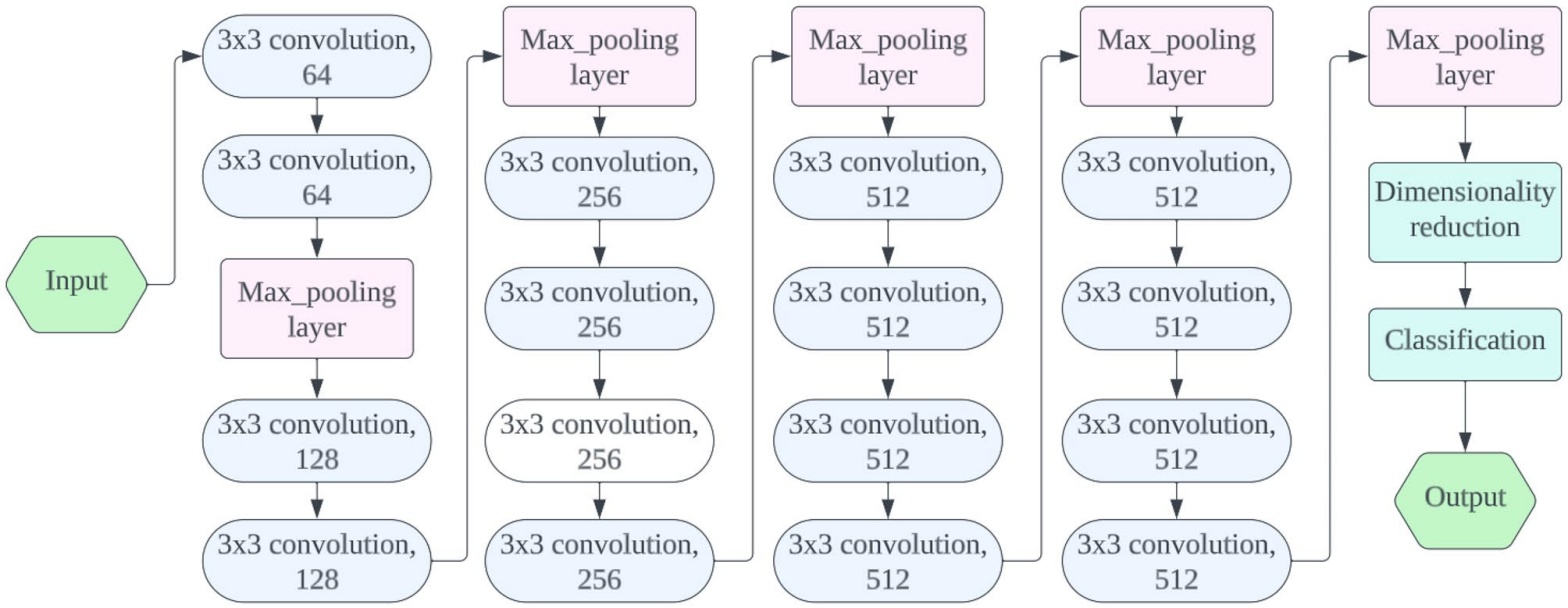
VGG19 architecture.

VGG19 is a deep convolutional neural network architecture, an evolution of the original VGG16 architecture. It consists of 19 layers, including 16 convolutional layers and 3 fully connected layers. VGG19 captures intricate patterns and features in image data through its deep architecture, which uses 3 × 3 convolutional filters for feature extraction^37^. Max-pooling layers reduce input spatial dimensions, lowering computational complexity. The final layers are fully connected, allowing predictions based on high-level features extracted by the convolutional layers. VGG19 uses the Rectified Linear Unit (ReLU) activation function for non-linearity. Widely used for image classification, VGG19 has become a benchmark in computer vision. Despite its depth and simplicity, it has been surpassed by modern architectures like ResNet and Inception in terms of performance and efficiency. Figure 5 depicts the architecture of VGG19.

##### DenseNet169

**Figure 6:**
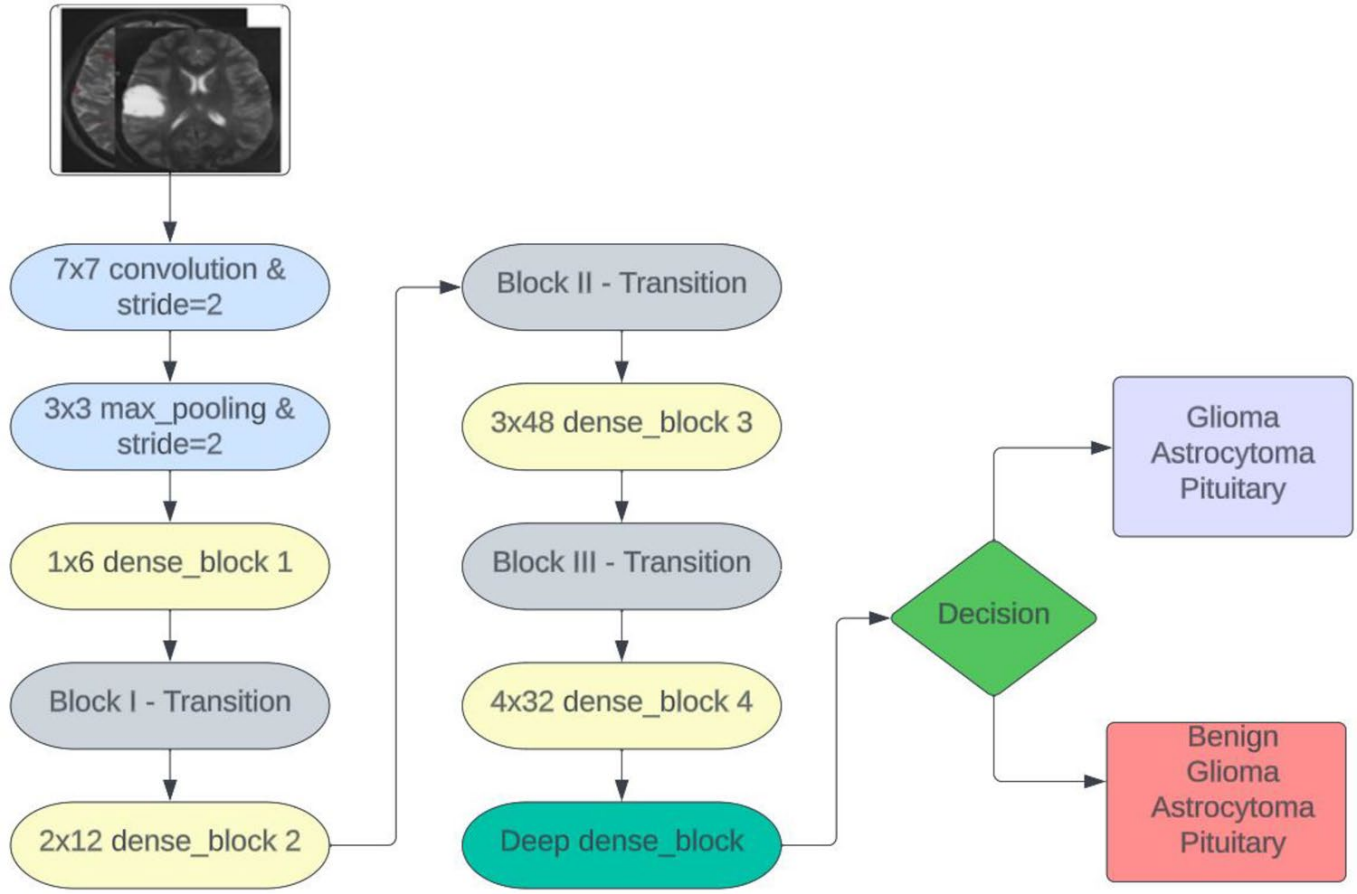
DenseNet169 architecture.

DenseNet169 is a convolutional neural network (CNN) architecture designed to overcome challenges in feature reuse and gradient flow in deep networks. Named after its 169 layers, it features dense connectivity, where each layer receives input from all preceding layers, promoting efficient feature reuse and enhanced information flow. To address computational complexity, DenseNet169 utilizes bottleneck layers, which incorporate 1 × 1 convolutions to reduce the number of input feature maps^38^. Dense blocks, each containing multiple densely connected layers, contribute to the overall depth and feature extraction capabilities. Transition layers are employed between dense blocks to control feature map growth and reduce spatial dimensions. DenseNet architectures commonly use global average pooling, which reduces the number of parameters and aids in better generalization. DenseNet169 has demonstrated strong performance in image classification tasks and is known for its parameter efficiency, achieving competitive accuracy with fewer parameters compared to other architectures. Figure 6 depicts the DenseNet169 architecture.

##### MobileNetv3

**Figure 7:**
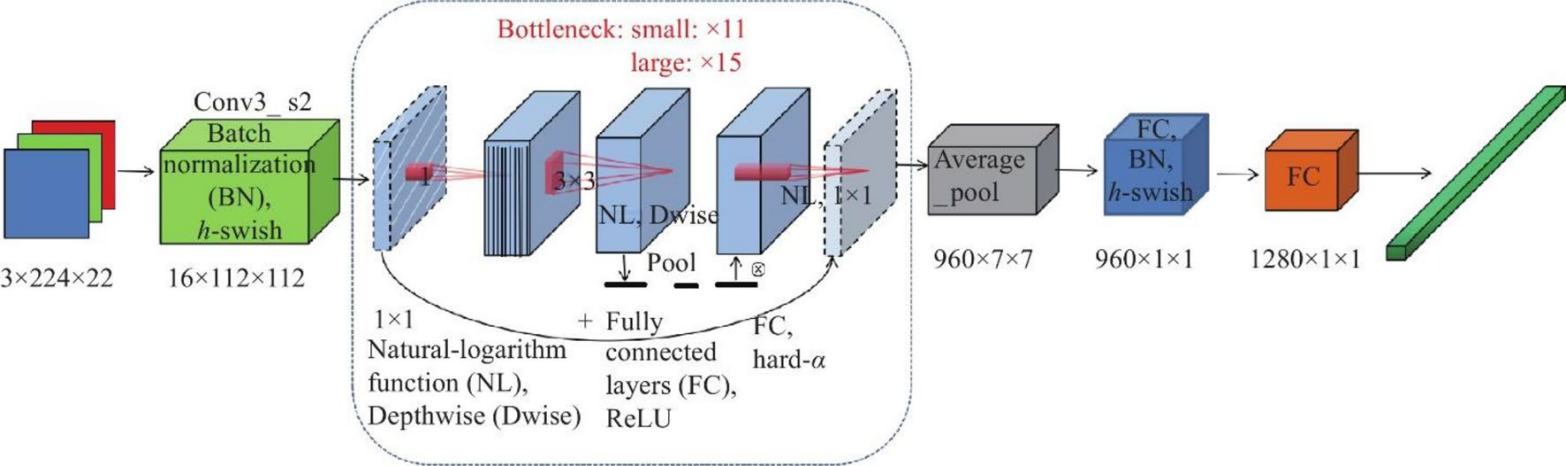
MobileNetv3 architecture.

MobileNetV3 is a neural network architecture designed for mobile and edge devices with limited computational resources. It is the third iteration of the MobileNet series, focusing on efficiency, speed, and accuracy. Key features include resource-efficient building blocks, lightweight inverted residuals, and two variants: MobileNetV3-Large and MobileNetV3-Small^39^. These building blocks optimize computation and memory usage, ensuring efficient operation on resource-limited hardware. Inverted residuals reduce computational overhead while maintaining the network's ability to extract meaningful features from input data. MobileNetV3 is available in two variants: MobileNetV3-Large for moderate computational resources and MobileNetV3-Small for strict constraints. Figure 7 depicts the architecture of MobileNetv3.

MobileNetV3 is a network architecture designed for tasks such as image classification, object detection, and semantic segmentation. It uses non-linear activation functions, such as swish and hard-swish, to improve model accuracy and learn more complex decision boundaries. Efficient Squeeze-and-Excitation (SE) blocks are used for channel-wise feature recalibration, enhancing the network's representational power. Techniques like neural architecture search and network pruning are employed to optimize the architecture for deployment on devices with limited resources. The lightweight design of MobileNetV3 makes it suitable for deployment on mobile devices, enabling real-time inference on edge devices with constrained resources. The architecture features a bottleneck block, allowing for dynamic adjustment of channel importance, enhancing performance. Table 3. Specification and bottle neck block of MobileNetv3.

**Table 3:** Specification and bottle neck block of MobileNetv3.

Input	Operator	Size	Output	Stride
224 × 224 × 3	conv2d, 3 × 3	–	16	2
112 × 112 × 16	bneck, 3 × 3	16	16	2
56 × 56 × 16	bneck, 3 × 3	72	24	2
28 × 28 × 24	bneck, 5 × 5	88	24	1
28 × 28 × 24	bneck, 5 × 5	96	40	2
14 × 14 × 40	bneck, 5 × 5	240	40	1
14 × 14 × 40	bneck, 5 × 5	240	40	1
14 × 14 × 40	bneck, 5 × 5	120	48	1
14 × 14 × 48	bneck, 5 × 5	144	48	1
14 × 14 × 48	bneck, 5 × 5	288	96	2
7 × 7 × 96	bneck, 5 × 5	576	96	1
7 × 7 × 96	bneck, 5 × 5	576	96	1
7 × 7 × 96	conv2d, 1 × 1	–	576	1
7 × 7 × 576	pool, 7 × 7	–	–	1
1 × 1 × 576	conv2d, 1 × 1, NBN	–	1024	1
1 × 1 × 1024	conv2d, 1 × 1, NBN	–	k	1

#### Preparation and evaluation of experiments

In this experiment, a large dataset of images was employed, and the training of our model was conducted on Google Colab. To ensure the effectiveness of the training and testing phases, access to a robust computing environment is essential. Kaggle was used to re-publish the dataset's training names. Importantly, the same dataset was utilized for all advanced models, encompassing both the training set and the test set. The Transfer Learning (TL) model underwent training using the specified training dataset and was subsequently evaluated using the corresponding test dataset. The success of our models can be attributed to the collaborative contributions of Sklearn, TensorFlow, and Keras. For optimal performance in all high-end models, a block size of 128 was determined to be the most effective. Table 4 illustrates the hyperparameter details of transfer learning models.

**Table 4:** Hyperparameters of transfer learning models for image classification.

Quantifying performance and evaluation	Assessing measurement outcomes
Size of the batch	128
Optimizer	Adam
No. of epochs	50
Rate of learning	0.001
Evaluation criterion	Cross entropy loss
Training	Five-fold cross validation

**Figure 8:**
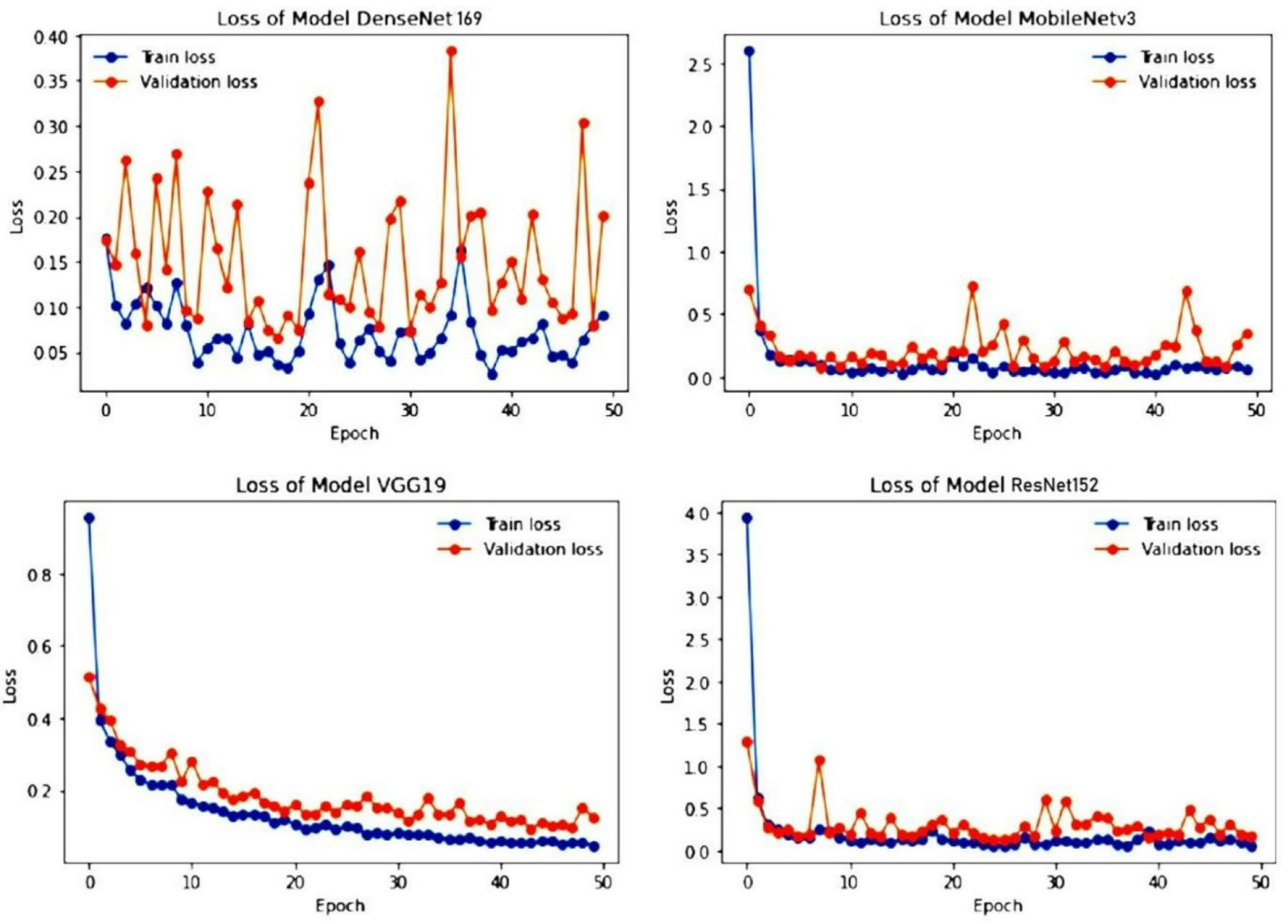
Training and testing loss of DenseNet169, MobileNetv3, VGG19 and ResNet152.

We applied the cross-entropy loss to both the train and test sets for each epoch. All models were trained for 50 epochs using the Adam optimizer with a learning rate of 0.001. Figure 8 shows the training and validation loss for each model over the course of the training epochs. For the ResNet152, VGG19, and MobileNetv3 models, the training and validation losses are very close and sometimes overlap. However, the DenseNet169 model exhibits a different behavior. While the training loss decreases, the validation loss increases for every epoch. This suggests that the DenseNet169 model may be overfitting the training data. In contrast, the MobileNetv3 model shows a stable training process with minimal fluctuations in both training and validation loss. By the end of training, the MobileNetv3 model achieves a training loss of 0.0451 and a validation loss of 0.1265. The VGG19 and ResNet152 models achieve training losses of 0.0603 and 0.001 and validation losses of 0.1862 and 0.010, respectively. Among the evaluated models, ResNet152 exhibited superior performance, achieving the highest validation loss of 0.0241 at epoch 39 and validation accuracy of 98.86%.

**Figure 9:**
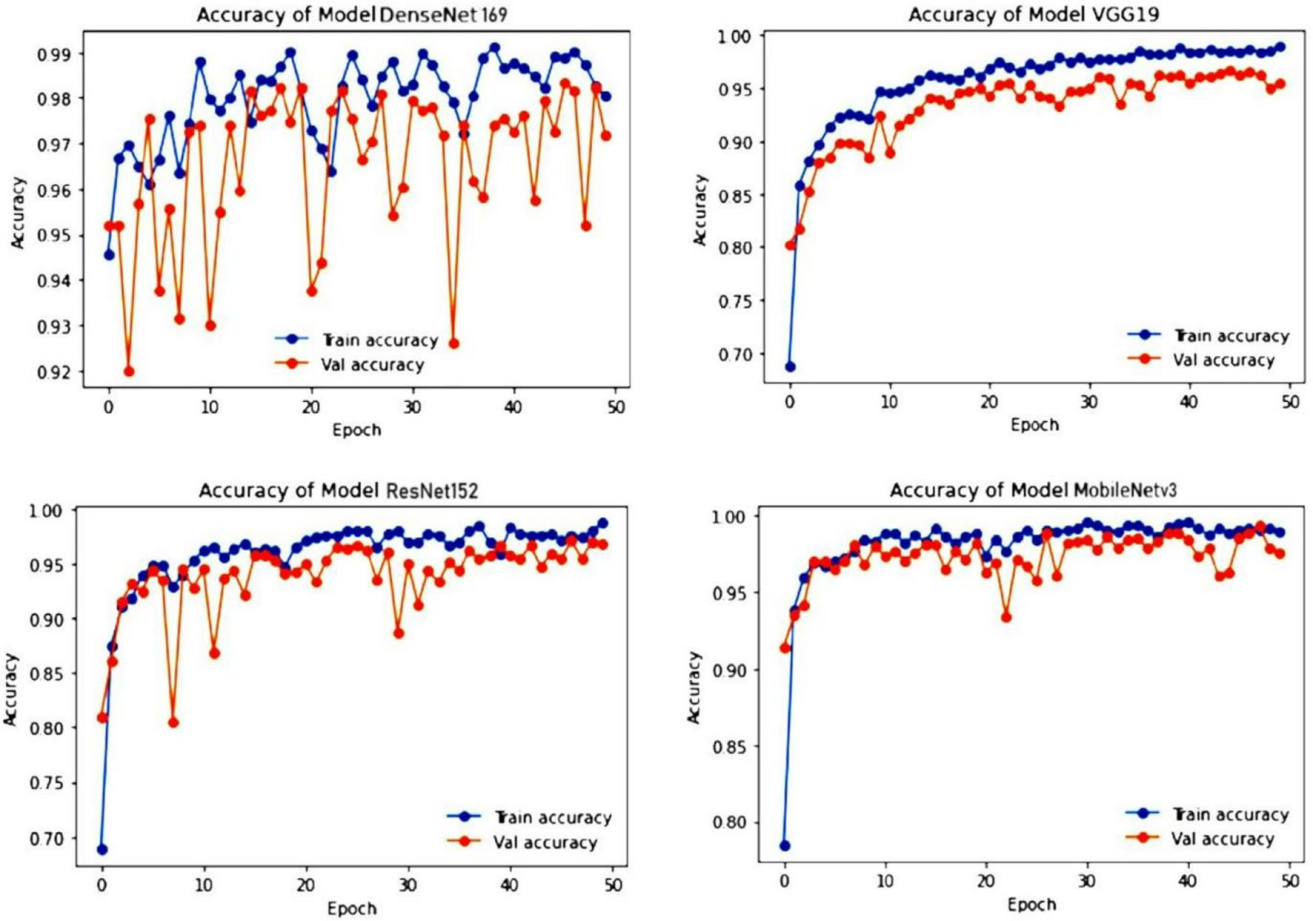
Training and testing accuracy of DenseNet169, MobileNetv3, VGG19 and ResNet152.

Among the evaluated models, DenseNet169 exhibited the lowest validation loss of 0.0664 at epoch 18 but experienced the most fluctuation in both training and validation accuracy. It ultimately achieved training and validation accuracies of 99.22% and 98.32%, respectively. VGG19 and MobileNetv3 also demonstrated promising results, with VGG19 achieving training and validation accuracies of 99.07% and 96.72%, respectively, and MobileNetv3 achieving training and validation accuracies of 99.75% and 98.52%, respectively. While VGG19's training and validation accuracies were relatively stable, MobileNetv3's validation accuracy showed some fluctuation. Figure 9 depicts the training and testing accuracy of four transfer learning models. Table 5, illustrates the four models training phase and testing phase accuracy and loss values.

**Table 5:** Performance across four transfer learning models Over 50 Epochs.

Architecture	Training phase		Testing phase	
	Acc (%)	Loss	Acc (%)	Loss
ResNet152	98.86	0.0603	96.92	0.1854
VGG19	99.07	0.0451	95.62	0.1245
DenseNet169	99.22	0.0241	97.53	0.958
MobileNetv3	99.75	0.0359	98.52	0.1272

## Experimental results and discussion

The proposed transfer learning models leverage the confusion matrix to assess their performance, employing metrics like precision, recall, F1 score, and accuracy. The confusion matrix, typically a square matrix, provides a comprehensive overview of model performance. Table 6 presents the confusion matrix, where TP denotes true positives, FP denotes false positives, and FN denotes false negatives. The F1 score is derived as the harmonic mean of precision and recall.

**Table 6:** Formula for confusion matrix.

	Predicted positive	Predicted negative
Actual positive	TP	FN
Actual negative	FP	TN

**Figure 10:**
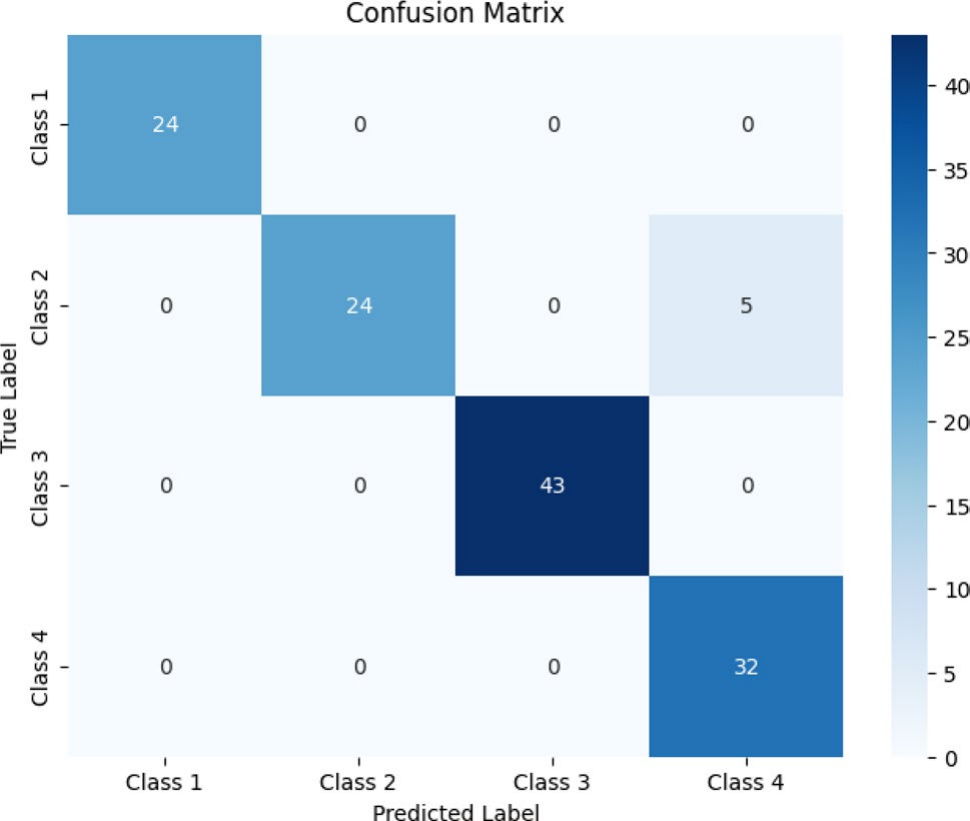
MobileNetv3 confusion matrix.

We conducted a comprehensive assessment of our model's performance, and the findings are presented through the examination of various performance metrics. Specifically, in Fig. 10, we depict the confusion matrix associated with the MobileNet model. The confusion matrix × serves as a valuable tool for evaluating the model's classification outcomes. In this matrix, the tumor classes are systematically labelled from 0 to 3, where each numerical identifier corresponds to a specific tumor type: 0 for 'Pituitary,' 1 for 'Normal,' 2 for 'Meningioma,' and 3 for 'Glioma.' This systematic numbering allows for a clear representation of the model's classification results. Upon close inspection of the confusion matrix, it is evident that the MobileNet model demonstrated commendable performance. Specifically, the model correctly identified 24 images belonging to the 'Pituitary' class, accurately classified 24 images as 'No tumor,' recognized 43 images as 'Meningioma,' and correctly identified 32 images as 'Glioma.'

**Figure 11:**
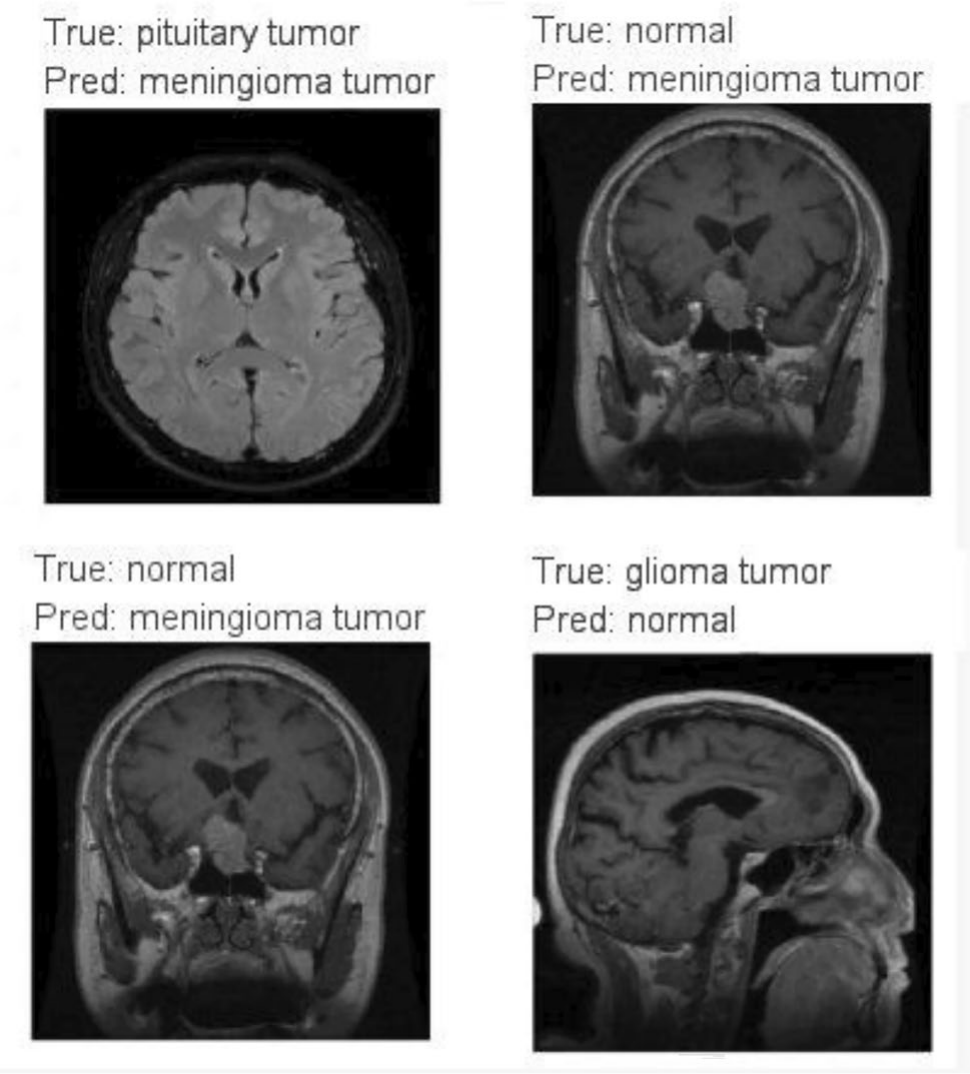
Incorrectly classified by proposed model.

Figure 11, depicts the incorrectly classified images by the proposed model. These numerical values within the matrix provide valuable insights into the model's effectiveness in accurately categorizing images across different tumor classes. Table 7 summarizes the performance metrics of four transfer learning models on the test dataset, providing insights into their efficacy in handling the given task. ResNet152 achieved the highest accuracy, followed by VGG19, DenseNet169, and MobileNetV3, which demonstrated commendable accuracy but trailed slightly behind. These evaluations were conducted after training each model for 50 epochs, suggesting that ResNet152 consistently outperformed the other models. The varying architectures of the four models underscore the nuanced trade-offs between computational efficiency and model accuracy. This analysis aids in discerning their strengths and weaknesses, offering valuable insights for informed decision-making.

**Table 7:** Five-fold test performance of four transfer learning models on the dataset.

Architectures	Class	Precision	Recall	F1 score	Accuracy
ResNet152	Pituitary	1	0.93	0.98	0.985
	Normal	0.98	1	0.97	
	Meningioma	1	1	1	
	Glioma	0.96	1	0.99	
	Total	3.94	3.93	3.94	
VGG19	Pituitary	1	1	0.95	0.960
	Normal	0.95	0.92	0.95	
	Meningioma	1	1	1	
	Glioma	0.93	1	0.94	
	Total	3.88	3.92	3.84	
DenseNet169	Pituitary	1	0.85	0.94	0.9675
	Normal	0.88	1	0.93	
	Meningioma	1	1	1	
	Glioma	1	1	1	
	Total	3.88	3.85	3.87	
MobileNetv3	Pituitary	1	1	1	0.960
	Normal	1	0.83	0.92	
	Meningioma	1	1	1	
	Glioma	0.88	1	0.92	
	Total	3.88	3.83	3.84	

### Discussion

Table 8 provides a comprehensive overview of the accuracy metrics for all models at both the maximum and minimum epoch numbers. The MobileNet model outperforms all other models, achieving the highest training accuracy of 99.75% at epoch 50 and a peak validation accuracy of 98.52% at epoch 45. To facilitate concise communication and notation, we use the terms Mx_Acc and Mi_Acc to represent Maximum Accuracy and Minimum Accuracy, respectively, and Mx_Ep and Mi_Ep to represent Maximum Epochs and Minimum Epochs, respectively. Table 9 details the duration of the training set for each epoch, providing insights into the time investment required for model training on Google Collaboratory's Graphics Processing Unit (GPU) runtime. This information is crucial for optimizing resource allocation and enhancing the efficiency of model training procedures.

**Table 8:** Accuracy summary across epochs for different transfer learning models.

Transfer learning model	Phase	M × -Acc	M × -Ep	Mi-Acc	Mi-Ep
VGG19	Training	98.78	50	70.22	1
	Testing	96.91	50	81.45	1
ResNet152	Training	98.12	50	69.34	1
	Testing	97.78	45	80.96	6
DenseNet169	Training	99.08	38	95.42	1
	Testing	98.68	47	90.77	2
MobileNetv3	Training	99.75	30	77.12	1
	Testing	99.52	46	90.27	1

**Table 9:** Epoch-wise training duration for transfer learning models.

TL models	Timeline (HH:MM)
ResNet152	03:15
VGG19	03:37
DenseNet169	02:45
MobileNetv3	04:33

**Figure 12:**
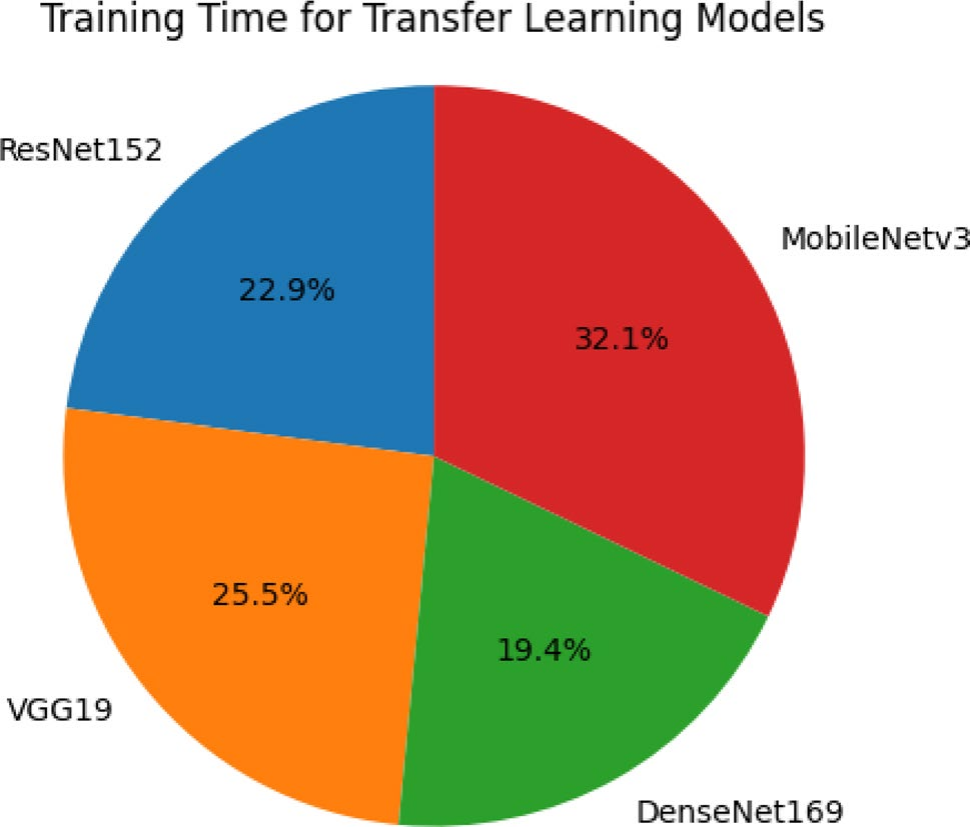
Training timeline for four transfer learning models.

**Figure 13:**
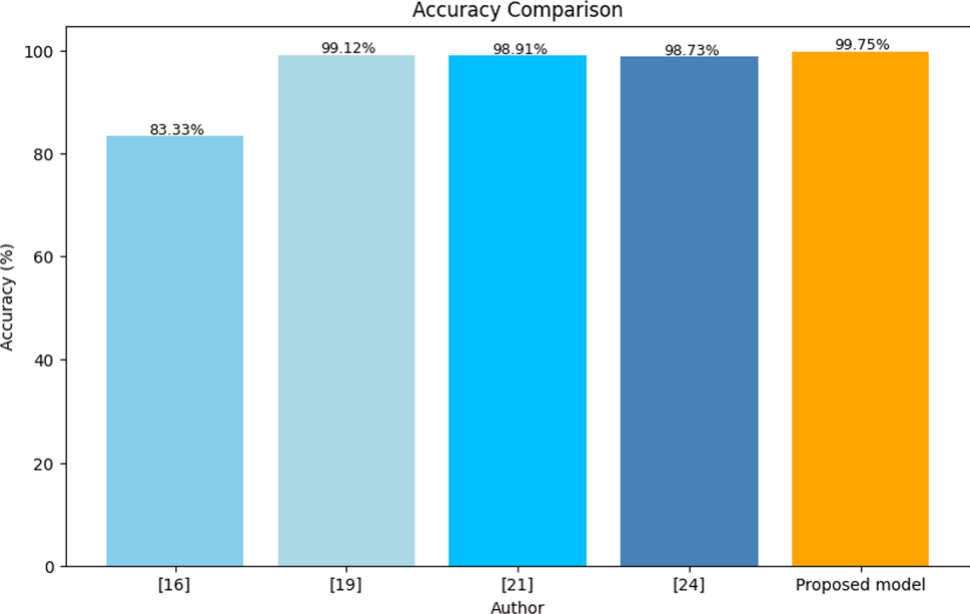
Accuracy comparison of proposed and state-of-the-art methods.

The precise detection and classification of brain tumors in medical images, particularly those obtained through MRI and CT scans, are crucial aspects of medical diagnostics. MRI is a powerful tool for medical diagnostics, playing a significant role in both diagnosing and categorizing various types of brain tumors. Table 8 and Fig. 12 summarizes the progress and anticipated future advancements in brain tumor detection and classification, comparing the current state with what we expect to achieve in the future. The MobileNetv3 model is a key part of our proposed approach and has achieved an impressive accuracy rate of 99.75%. This shows that the model is effective at discerning and predicting the presence of brain tumor cells in medical images, making it a valuable tool for medical diagnosis. Table 10 illustrates the classification accuracy comparison of the proposed and other existing models. Figure 12 depicts the classification accuracy comparison of proposed and other state-of-the-art methods. Figure 13 clearly demonstrates that the proposed model outperformed other models with an accuracy of 99.75%.

**Table 10:** Classification accuracy comparison of the proposed and other existing models.

Author	Year	Dataset	Method	Accuracy (%)
Tasnim Azad Abir^16^	2018	Kaggle	PNN	83.33
Bakary Badjie^19^	2022	Kaggle	AlexNet's CNN	99.12
Naeem Ullah^21^	2022	Kaggle	Inceptionresnetv2	98.91
Saravanan^24^	2020	Kaggle	CANFES	98.73
Proposed model	2023	Kaggle	Transfer Learning approach	99.75

## Conclusion and future work

In this investigation, we delve into the application of transfer learning methods for the classification of brain tumors using MRI scans. The study meticulously assesses the efficacy of four distinct transfer learning models—ResNet152, VGG19, DenseNet169, and MobileNetv3—across three diverse brain tumor image datasets. The evaluation encompasses crucial performance metrics, including accuracy, precision, f1-score, and recall. Remarkably, ResNet152 emerges as the frontrunner among the models, demonstrating outstanding performance with an accuracy of 98.5%, surpassing the performance of all other models in the study. Additionally, MobileNetv3 demonstrates exceptional efficacy with an accuracy of 99.75%, showcasing its robust performance in brain tumor classification. It's important to highlight that this study relies on a secondary dataset. Future research could explore extending the proposed model's application to CT images, enhancing its adaptability. This extension holds the potential to broaden the model's impact in medical applications. In conclusion, the proposed model, particularly ResNet152 and MobileNetv3, shows significant promise in advancing medical image classification. Continued investigation and exploration, involving a range of imaging modalities, offer the potential to uncover valuable insights that could significantly enhance applications in medical diagnostics. By expanding the study to include diverse imaging techniques beyond the currently examined MRI scans—such as PET, CT, or ultrasound—researchers can achieve a more comprehensive understanding of the proposed model's adaptability and effectiveness across a broader spectrum of medical imaging data. The integration of diverse imaging modalities not only allows for a more holistic assessment of the proposed model's performance but also contributes to its resilience and applicability in real-world medical scenarios. Each imaging modality comes with unique challenges and characteristics, and a collective exploration can refine the proposed model's capabilities while pinpointing areas for improvement. The study has some limitations. It didn't check how well the model works in different situations or on other datasets, making it unclear how it would perform in the real world. The dataset used might not represent all kinds of patients, causing potential biases in the predictions. The study also didn't consider the costs of training and using the models, which could be a problem for using them in healthcare. Despite these limitations, future work will aim to improve the model's usefulness in different healthcare settings. In the future, we aim to explore more model architectures, improve their performance, and make them better suited for different datasets and clinical situations. Ongoing research will focus on checking how well these models work in the real world by testing them on different datasets and in various clinical settings. Additionally, we plan to refine image enhancement techniques for specific tumor categories to ensure a well-balanced dataset and robust models.

## Data Availability

We used the balanced dataset which is publicly available https://www.kaggle.com/datasets/masoudnickparvar/brain-tumor-mri-dataset
